# Prognostic factors analysis of surgical resection after conversion therapy for isolated pleural metastatic lung cancer: a retrospective analysis

**DOI:** 10.3389/fmed.2026.1859804

**Published:** 2026-06-19

**Authors:** Huan-Wei Qu, Shu-Han Liu, Ying Liu, Fen Han, Qiu-Yue Liu

**Affiliations:** Surgical Intensive Care Unit, Beijing Chest Hospital, Capital Medical University, Beijing Tuberculosis and Thoracic Tumor Research Institute, Beijing, China

**Keywords:** conversion therapy, lung cancer, pleural metastasis, prognostic factors, surgical resection

## Abstract

**Objective:**

To explore the efficacy and prognostic factors of surgical resection in patients with non-small cell lung cancer (NSCLC) and isolated pleural metastasis (M1a) after receiving individualized conversion therapy.

**Methods:**

This retrospective study included 167 patients with non-small cell lung carcinoma (NSCLC) and isolated pleural metastasis (M1a) admitted to Beijing Chest Hospital, Capital Medical University, from January 2019 to December 2023. Based on genotyping results, targeted therapy or chemo-immunotherapy regimens were administered. After conversion therapy, a multidisciplinary team (MDT) assessed surgical feasibility. The primary endpoint following comprehensive treatment was overall survival (OS) and progression-free survival (PFS), secondary endpoints included Objective response rate (ORR) and disease control rate (DCR); incidence of postoperative complications and Recurrence status. Survival probabilities were estimated using the Kaplan–Meier method, and independent prognostic factors were identified using the Cox proportional hazards regression model.

**Results:**

The median follow-up for all patients was 28.5 months. In total, 167 patients (82.26%) underwent curative or cytoreductive surgery. The median OS was 22.6 months (95%CI: 18.4–26.8), and the median PFS was 12.4 months (95% CI: 9.2–15.6). The 1-year and 2-year survival rates were 89.7 and 42.1%, respectively. Multivariate analysis revealed that squamous cell carcinoma (vs. adenocarcinoma, HR = 1.68, *p* = 0.012), driver gene negativity (HR = 1.61, *p* = 0.024), SD as the response to conversion therapy (vs.PR/CR, HR = 1.82, *p* = 0.006), N2 lymph node metastasis (HR = 1.85, *p* = 0.003), and non-R0 resection (HR = 2.08, *p* = 0.001) were independent risk factors for OS. The postoperative complication rate was 12.57% (21/167); complications of Clavien-Dindo grade ≥3 occurred in 28.57% (6/21) of patients. Perioperative death occurred in one patient.

**Conclusion:**

Surgical resection after effective conversion therapy significantly improves long-term survival in patients with isolated pleural metastatic lung cancer. Histological subtype, molecular characteristics, and completeness of resection are key prognostic indicators. Clinically, surgical intervention should prioritize patients who demonstrate a favorable response to conversion therapy, achieve R0 resection, and harbor driver gene mutations.

**Clinical trial registration:**

http://www.chictr.org.cn/, identifier ChiCTR2200056791.

## Background

Lung cancer is one of the most prevalent and lethal malignant tumors globally, representing a significant global health burden (1). According to the IASLC 8th edition TNM staging system, pleural metastasis (M1a) is classified as stage IV lung cancer and was historically considered an absolute contraindication for surgical treatment (2). Traditional therapies primarily consist of systemic chemotherapy, targeted therapy, or immunotherapy, but overall prognosis remains poor, with a median overall survival (OS) typically ranging from 6 to 12 months (3). In recent years, the rapid development of immune checkpoint inhibitors, targeted therapies, and anti-angiogenic drugs has significantly improved treatment outcomes for advanced lung cancer (4). Previous studies have shown that the objective response rate (ORR) for immunotherapy combined with chemotherapy or anti-angiogenic therapy can reach 40–60%, with most patients achieving a complete or near-complete radiographic response (5). This breakthrough has created opportunities for conversion therapy, where unresectable tumors are downstaged to resectability through comprehensive treatment, enabling subsequent surgical resection (6, 7).

For lung cancer patients with isolated or localized pleural metastases, surgical resection after effective conversion therapy and stable disease control has emerged as an emerging therapeutic strategy gaining global attention (8). Carefully selected patients undergoing surgical treatment demonstrate better long-term survival outcomes compared to systemic therapy alone. However, current research in this area remains limited, with unclear prognostic factors and a lack of standardized patient selection criteria and treatment strategies.

In this retrospective study, we reviewed the clinical data of patients diagnosed with isolated pleural metastatic lung cancer who underwent surgical excision after conversion therapy at our hospital over the past four years. This study aimed to identify key factors influencing postoperative prognosis to provide evidence-based support for clinical screening of suitable surgical candidates and optimizing perioperative management.

## Materials and methods

### General information

**Figure 1:**
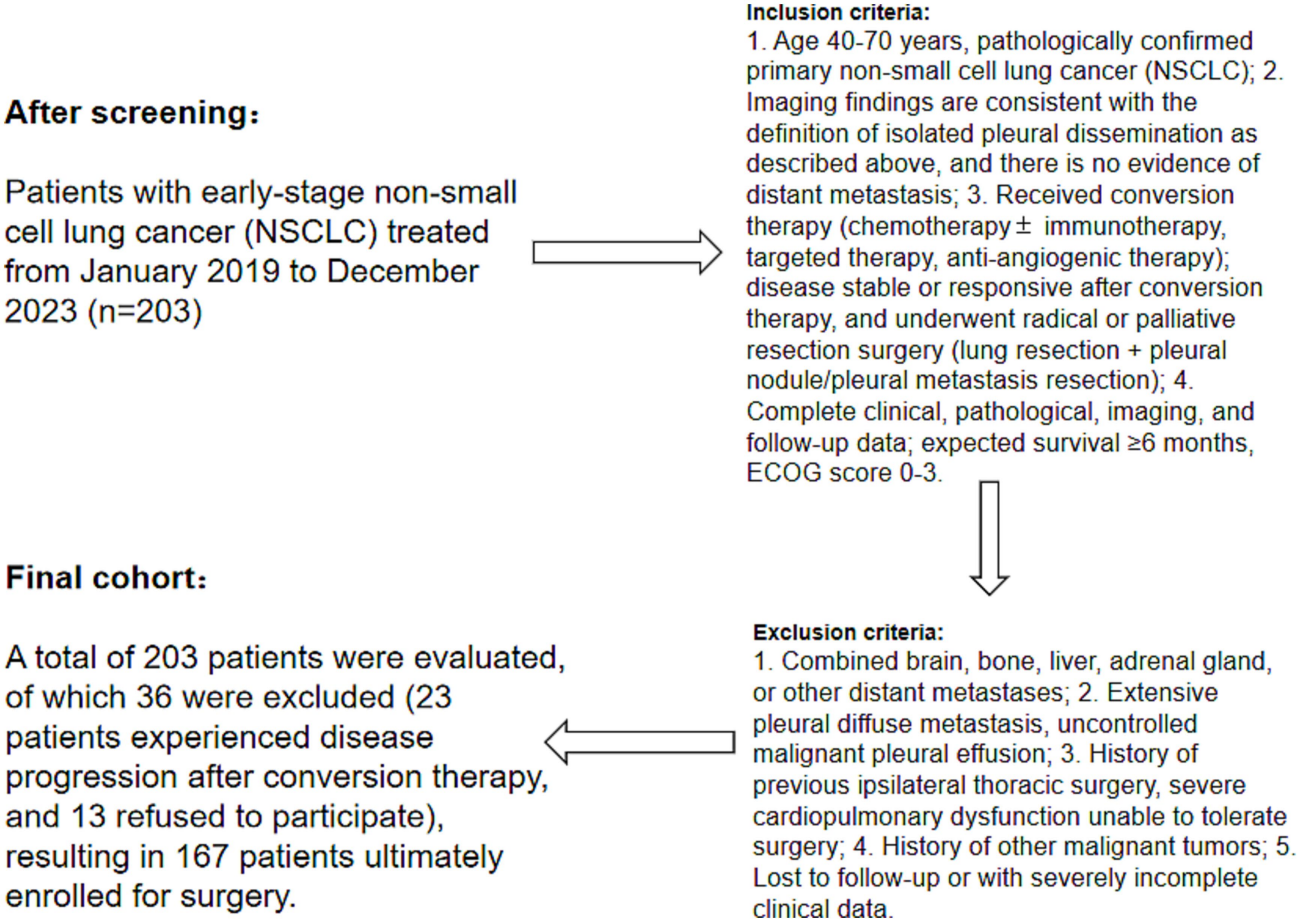
The flowchart illustrates the complete screening process.

A retrospective analysis was conducted on the clinical data of patients with isolated pleural metastatic lung cancer who underwent surgical resection after conversion therapy at Beijing Chest Hospital, Capital Medical University, from January 2019 to December 2023. In this study, isolated pleural dissemination was defined as imaging findings demonstrating well-defined isolated nodular pleural thickening or distinct pleural nodules lacking partial or extensive fusion, irrespective of nodule count or the presence of pleural effusion, all considered as isolated nodules. Inclusion criteria: 1. age 40–70 years, pathologically confirmed as primary non-small cell lung cancer (NSCLC); 2. imaging findings are consistent with the definition of isolated pleural dissemination as described above, and there is no evidence of distant metastasis; 3. Received conversion therapy (chemotherapy ± immunotherapy, targeted therapy, anti-angiogenic therapy); after conversion therapy, the disease was stable or relieved, and radical or palliative resection surgery (lung resection + pleural nodule/pleural metastasis resection) was performed; 4. Complete clinical, pathological, imaging, and follow-up data; expected survival ≥6 months, ECOG score 0–3. Exclusion criteria: 1. presence of other distant metastases such as brain, bone, liver, or adrenal gland; 2. extensive diffuse pleural metastasis, uncontrolled malignant pleural effusion (MPE); 3. previous major ipsilateral thoracic surgeries (such as lobectomy, pneumonectomy, extensive pleurectomy), severe cardiopulmonary dysfunction unable to tolerate surgery; 4. History of other malignant tumors; 5. Loss to follow-up or insufficient clinical data. This study was approved by the hospital ethics committee (approval number: LW-2019-017), and all patients provided written informed consent. We declare that all research procedures were conducted in accordance with the Declaration of Helsinki. The exact process is shown in our flowchart (Figure 1).

### Conversion treatment plan

Develop an individualized conversion treatment plan based on the patient’s molecular characteristics. Patients who are negative for driver genes receive chemotherapy combined with immune-checkpoint inhibitors (PD-1/PD-L1 inhibitors) ± anti-angiogenic therapy; patients who are positive for driver genes (EGFR/ALK/ROS1, etc.) receive corresponding targeted drugs±chemotherapy. Efficacy evaluation is performed every 2 cycles, using the Response Evaluation Criteria in Solid Tumors (RECIST 1.1) for efficacy assessment. Postoperatively, adjuvant therapy is administered based on the pathological findings.

### Surgical treatment

After effective conversion therapy or disease stabilization, the feasibility of surgery is assessed by a multi-disciplinary team (MDT). The multi-disciplinary team confirms that the patient has successfully achieved downstaging and is eligible for surgery based on the following criteria: (1) At least stable disease or partial/complete response according to RECIST 1.1 criteria; (2) Imaging demonstrates that the primary tumor is resectable with clear anatomical margins; (3) The pleural effusion has resolved or significantly decreased; (4) No new distant metastases; (5) Good performance status (ECOG ≤2) and sufficient cardiopulmonary reserve to tolerate surgery. Surgical methods include lobectomy, wedge resection, or lung segmental resection combined with resection of pleural metastases, pleural decortication, or hyperthermic intrathoracic chemotherapy (HITHOC). Throughout the surgical procedure, oncological principles must be strictly observed (except for palliative surgery), including: (1) formulating a standardized lymph node dissection plan according to the International Association for the Study of Lung Cancer (IASLC) guidelines; (2) maintaining a surgical margin of at least 2 cm or the maximum tumor dimension (whichever is larger), with intraoperative frozen section analysis to confirm negative margins; (3) preserving key vascular and bronchial structures to ensure complete tumor resection while minimizing functional impairment; (4) employing three-dimensional high-definition thoracoscopy intraoperatively to ensure surgical precision. Surgical completeness is classified based on intraoperative frozen section analysis as R0 resection (microscopically negative margin) and R1 resection (microscopically positive margin). The surgical principle is to achieve complete resection of the primary tumor and pleural metastases to the greatest extent feasible, with the goal of achieving the R0 resection criteria.

### Follow-up plan

All enrolled patients were evaluated at 1 month postoperatively, followed by assessments every 3 months in the first year, every 6 months for the subsequent 2 years, and annually thereafter. During follow-up, routine chest and abdomen computed tomography (CT), as well as brain CT or magnetic resonance imaging (MRI) scans were performed. Single-photon emission computed tomography (SPECT) bone scans were performed annually, and positron emission tomography-computed tomography (PET-CT) was performed when necessary. If outpatient visits could not be arranged, telephone follow-up was conducted. Follow-up for each patient continued until death or December 2023, whichever occurred first.

### Observation indicators and endpoints

Primary endpoint include Overall survival (OS):defined as the time from the date of surgery to all-cause death or the last follow-up and Progression-free survival (PFS): the time from the date of surgery to disease recurrence, progression, or death; Secondary endpoints include Objective response rate (ORR) and disease control rate (DCR); Incidence of postoperative complications and incidence of severe complications (Clavien-Dindo classification≥3 grade); Recurrence status: local recurrence, regional recurrence, distant metastasis.

### Statistical methods

Data analysis was performed using SPSS 26.0 statistical software. Continuous variables are expressed as mean ± standard deviation or median (interquartile range), and categorical variables are expressed as number of cases (percentage). Survival curves were plotted using the Kaplan–Meier method, and differences in survival between groups were compared using the Log-rank test. Potential prognostic factors were identified via univariate analysis, and variables with a *p*-value <0.1, or those considered clinically important based on previous literature and expert knowledge, were included in the multivariate model. The proportional hazards assumption was assessed using Schoenfeld residuals, and variables violating this assumption (Schoenfeld test *p* < 0.05) were addressed via stratified analysis. Comparisons of categorical variables were performed using the χ^2^ test or Fisher’s exact test. A two-sided *p* < 0.05 was considered statistically significant.

## Results

### Baseline characteristics of patients

A total of 167 patients were included, comprising 90 males (53.89%) and 77 females (46.11%). The median age was 58 years (range: 40–70 years). The predominant pathological type was adenocarcinoma (124 cases, 74.25%), with squamous cell carcinoma in 43 cases (25.74%). There were 113 patients (67.66%) with positive driver genes, including 71 cases (62.83%) with EGFR mutations, 15 cases (13.27%) with ALK fusions, 16 cases (14.15%) with ROS1 fusions, 6 cases (5.32%) with KRAS gene mutation, and 5 cases (4.42%) with BRAF gene mutation. There were 54 patients (32.33%) with non-driver gene positivity. PD-L1 expression was positive (TPS ≥ 1%) in 54 cases (100%). The median number of cycles in conversion therapy was 4 cycles (range: 2–8 cycles). Detailed basic information of patients is shown in Table 1.

**Table 1:** The baseline characteristics of the patients are detailed.

Variables	*N*	%
Histology		
Gender		
Male	90	53.89
Female	77	46.11
Lung squamous cell carcinoma	43	25.74
Lung adenocarcinoma	124	74.25
Age		
<65 years old	97	58.08
≥65 years old	70	41.91
Smoking history		
Yes	107	64.07
No	60	35.93
ECOG performance status score		
0–1 Score	119	71.26
2–3 Score	48	28.74
Molecular characteristics		
Non-driver gene positive (PD-1/PD-L1 inhibitors)	54	32.34
Driver gene positive (EGFR/ALK/ROS1)	113	67.66
Body mass index (BMI, kg/m^2^)		
<18.5	45	26.94
18.5 ~ 23.9	91	54.49
≥24.0	31	18.56
Comorbidities		
Hypertension	42	25.14
Diabetes mellitus	38	22.75
Mild coronary artery disease	25	14.97
Chronic obstructive pulmonary disease	57	34.13
Pulmonary function (FEV1 forced expiratory volume in the first second)		
≥80%	100	59.88
<80%	67	40.12
Pulmonary function (FEV1/FVC)		
≥0.7	98	58.68
<0.7	69	41.32

### Efficacy of conversion therapy

The efficacy of conversion therapy was evaluated according to RECIST 1.1 criteria (9): complete response (CR) in 49 cases (29.34%), partial response (PR) in 62 cases (37.12%), stable disease (SD) in 33 cases (19.76%), and disease progression (PD) in 23 cases (13.77%). The objective response rate (ORR) was 66.46% (111/167), and the disease control rate (DCR) was 86.22% (144/167). The curative effect evaluation of enrolled patients after conversion therapy is detailed in Table 2.

**Table 2:** The efficacy evaluation after conversion therapy.

Group	Complete response (CR) (%)	Partial response (PR) (%)	Stable disease (SD) (%)	Progressive disease (PD) (%)	Objective response rate (ORR) (%)	Disease control rate (DCR) (%)
RECIST criteria						
*n*	49 (29.34%)	62 (37.12%)	33 (19.76%)	23 (13.77%)	111 (66.46%)	144 (86.22%)

### Surgical conditions

The surgical rate was 82.26% (167/203), with 23 patients not undergoing surgery due to disease progression, 13 patient abandoning surgical treatment, and the remaining 167 patients all receiving surgery. Surgical methods: Lobectomy (Pulmonary lobectomy) in 105 cases (62.87%), Lung segmental resection in 33 cases (19.76%), and wedge resection in 9 cases (5.38%). Combined resection of pleural metastatic lesions in 70 cases (41.91%), pleural decortication in 52 cases (31.13%), and hyperthermic intrathoracic chemotherapy (HITHOC) in 18 cases (10.77%). Surgical radicality: R0 resection in 144 cases (86.22%), R1 resection in 23 cases (13.77%). The median Operative time was 165 min (range: 90–280 min), and the median blood loss was 200 mL (range: 50–800 mL). Details are shown in Table 3.

**Table 3:** The surgical details of the enrolled patients.

Surgical method	*n*	%
Lobectomy	105	62.87
Segmental resection	33	19.76
Wedge resection	9	5.38
Combined resection of pleural metastatic lesions	70	41.91
Pleural decortication	52	31.13
Hyperthermic Intrathoracic Chemotherapy (HITHOC)	18	10.77
Surgical thoroughness		
R0 resection	144	86.22
R1 resection	23	13.77
Intraoperative indicators		
Operation time (min)	165	90–280 (min)
Blood loss (mL)	200	50–800(mL)
Total number of lymph nodes removed		
<10	42	25.14
≥10	125	74.85

### Postoperative complications

The incidence of postoperative complications was 12.57% (21/167), including 9 cases of lung infection (5.38%), 5 cases of arrhythmias (2.99%), 5 cases of pleural effusion requiring continuous drainage of >100 mL for >7 days (2.99%), 1 case of incision infection (0.59%), and 1 case of bronchopleural fistula (0.59%). A total of 6 cases (28.57%) developed Clavien-Dindo grade ≥3 complications of these, 2 required reoperation (9.52%) and 3 required transfer to the intensive care unit (14.28%). One perioperative death occurred (4.76%) (see Table 4).

**Table 4:** The postoperative complications of the enrolled patients.

Postoperative complications	*n*	%
Total postoperative complications	21	12.57
Lung infection	9	5.38
Arrhythmia	5	2.99
Persistent pleural effusion drainage of 100 ml for >7 days	5	2.99
Incision infection	1	0.59
Bronchopleural fistula	1	0.59
Clavien-Dindo classification		
< 3 classification	15	71.42
≥ 3 classification	6	28.57
Reoperation	2	9.52
Transferred to ICU	3	14.28
Perioperative death	1	4.76

### Survival analysis

**Figure 2:**
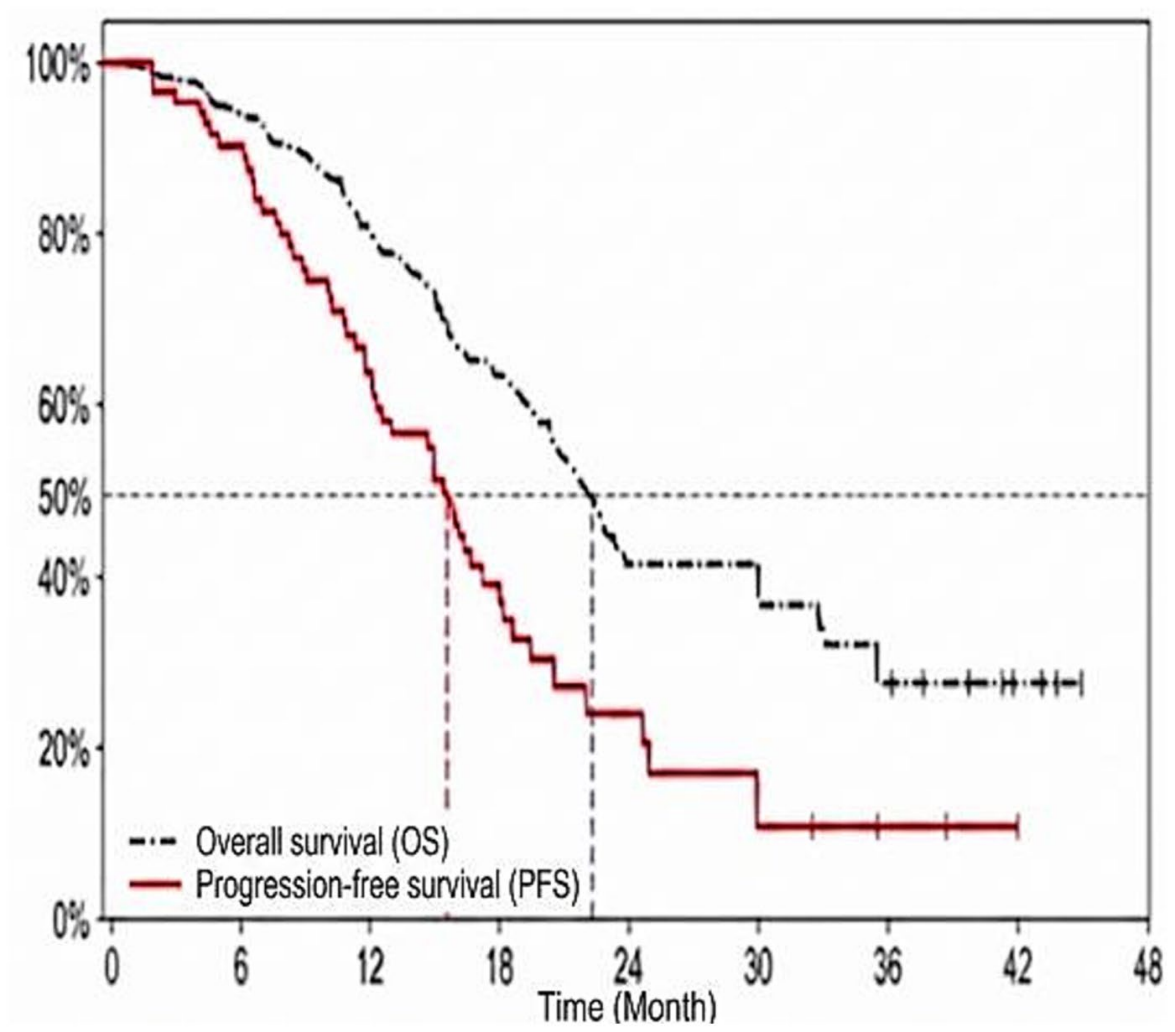
Overall survival (OS) and progression-free survival (PFS) of patients.

**Figure 3:**
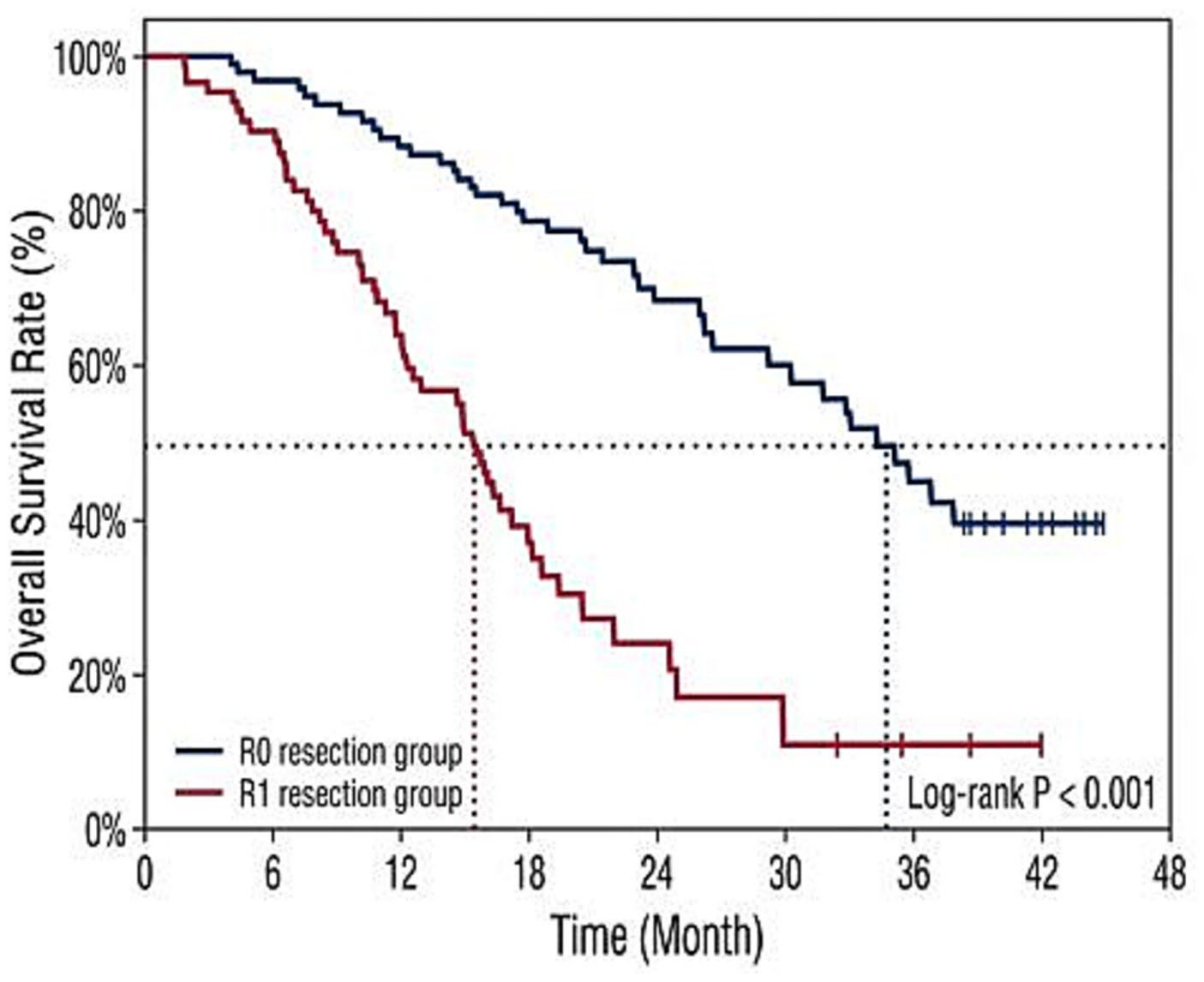
Comparison of overall survival rates between the R0 and R1 resection groups.

The median follow-up time was 28.5 months (range: 6.2–44.4 months). The median overall survival (OS) for the entire group was 22.6 months (95% CI: 18.4–26.8), and the median progression-free survival (PFS) was 12.4 months (95% CI: 9.2–15.6). The 1-year and 2-year survival rates were 89.7 and 42.1% in Figure 2, respectively. The median OS in the R0 resection group was significantly better than that in the R1 resection group (35.2 months vs. 15.8 months, *p* < 0.001) Figure 3.

### Univariate analysis

Univariate analysis showed that pathologic type, driver gene status, conversion therapy efficacy, lymph node involvement status, surgical radicality, and number of pleural metastases were significantly associated with OS (*p* < 0.05), as detailed in Table 5.

**Table 5:** Survival analysis of patients in different subgroups.

Subgroup	*n*	Median OS (months)	HR	95%CI	*p*-value
Pathological type					0.018
Adenocarcinoma	124	34.6	Ref		
Squamous cell carcinoma	43	21.7	1.72	1.10–2.68	
Driver gene status					0.031
Positive	113	34.5	Ref		
Negative	54	21.3	1.65	1.08–2.50	
Conversion therapy efficacy					0.008
PR/CR	111	35.2	Ref		
SD	33	22.2	1.85	1.20–2.85	
lymph node metastasis					0.005
N0/N1	32	34.4	Ref		
N2	135	22.5	1.88	1.25–2.82	
Surgical thoroughness					<0.001
R0	144	35.2	Ref		
R1	23	15.8	2.25	1.38–3.65	

### Multivariate analysis

Multivariate Cox regression analysis revealed that pathologic type (squamous cell carcinoma reference: adenocarcinoma, HR = 1.68, 95% CI: 1.12–2.52, *p* = 0.012), driver gene status (positive reference: negative, HR = 0.62, 95% CI: 0.41–0.94, *p* = 0.024), conversion therapy efficacy (PR/CR reference: SD, HR = 0.55, 95% CI: 0.36–0.84, *p* = 0.006), lymph node involvement status (N2 reference: N0/N1, HR = 1.85, 95%CI: 1.23–2.78, *p* = 0.003), and surgical radicality (R0 reference: R1, HR = 0.48, 95%CI: 0.31–0.74, *p* = 0.001) were independent prognostic factors affecting OS (see Table 6).

**Table 6:** The results of the multivariate cox regression analysis.

*N*	HR	95%CI	*p*-value
Pathological type (squamous cell carcinoma reference: adenocarcinoma)	1.68	1.12–2.52	0.012
Driver genes (positive reference: negative)	0.62	0.41–0.94	0.024
Therapeutic efficacy (PR/CR reference: SD)	0.55	0.36–0.84	0.006
Lymph nodes (N2 reference: N0/N1)	1.85	1.23–2.78	0.003
Surgical radicality (R0 reference: R1)	0.48	0.31–0.74	0.001

### Recurrence pattern

At the end of follow-up, a total of 7 patients died, and 35 patients (20.95%) experienced disease recurrence or progression. The recurrence patterns comprised 22 cases (13.17%) of locoregional recurrence and 13 cases (7.78%) of distant metastasis. Sites of distant metastasis: 5 cases in the contralateral lung, 3 in the bone, 2 in the brain, 2 in the liver, and 1 in the adrenal gland.

## Discussion

This single-center retrospective study of 167 patients with isolated pleural metastatic non-small cell lung cancer (NSCLC) who underwent surgical resection after individualized conversion therapy demonstrates the significant survival benefit of this strategy. The median follow-up for the entire cohort was 28.5 months, with a median overall survival (OS) of 22.6 months (95% CI: 18.4–26.8) and a median progression-free survival (PFS) of 12.4 months (95% CI: 9.2–15.6). The 1-year and 2-year survival rates were 89.7 and 42.1%, respectively, supporting the treatment model of conversion therapy combined with surgery in strictly selected patients. The objective response rate (ORR) of conversion therapy was 66.46%, and the disease control rate (DCR) was 86.22%, with 111 patients (66.46%) achieving partial response (PR) or complete response (CR), facilitating subsequent surgical resection. The application of immune-checkpoint inhibitors combined with chemotherapy, anti-angiogenic therapy, and targeted drugs significantly improved the treatment efficacy of advanced lung cancer, converting some previously unresectable patients into a resectable state. In this study, 167 patients ultimately underwent radical surgery or cytoreductive surgery, with a surgical rate of 82.26%. Through multivariate Cox proportional hazards regression analysis, histological subtype, driver gene status, imaging efficacy of conversion therapy, N2 lymph node metastasis status, and the extent of surgical radicality were precisely identified as independent prognostic factors determining OS. These findings provide a solid evidence-based medical foundation for optimizing perioperative treatment strategies and establishing surgical indications for patients with stage M1a NSCLC.

Multivariate analysis showed that pathologic type was an independent risk factor affecting overall survival (OS) (HR = 1.68, *p* = 0.012). This difference may be attributed to the following factors: First, the proportion of driver gene mutations in adenocarcinoma of the lung is relatively high (in this study, the prevalence of driver gene mutations such as epidermal growth factor receptor [EGFR], anaplastic lymphoma kinase [ALK], and ROS proto-oncogene 1, receptor tyrosine kinase [ROS1] reached 67.66%), which can benefit from targeted therapy; after receiving specific tyrosine kinase inhibitors (TKIs) treatment, these patients often achieve rapid, deep, and sustained cytotoxic effects on tumor cells, effectively clearing free cancer cells and microscopic seeding nodules in the pleural cavity, creating highly favorable anatomical downstaging conditions for subsequent surgery (10, 11). In contrast, the molecular profile of lung squamous cell carcinoma lacks such highly effective targetable driver genes, and its conversion therapy mainly relies on platinum-based doublet chemotherapy combined with immune-checkpoint inhibitors (programmed cell death protein 1 [PD-1]/programmed death-ligand 1 [PD-L1] monoclonal antibodies). When facing the special immune-privileged microenvironment of the malignant pleural cavity, its efficacy is significantly limited (12). Malignant pleural metastases and pleural effusions are rich in highly immunosuppressive cytokines such as transforming growth factor-beta (TGF-*β*) and interleukin-10 (IL-10). These cytokines form a biochemical barrier that severely hinders the infiltration and activation of effector CD8 + T cells, leading to a significant reduction in the efficacy of PD-1 blockade therapy in the local pleural cavity (13).

N2 lymph node metastasis is an independent risk factor associated with unfavorable prognosis. Previous studies have shown that the median overall survival (OS) of patients with N2 lymph node metastasis was reported as 22.8 months, significantly lower than the 35.4 months observed in patients with N0/N1 disease (14), these findings align with the results of the current study. This finding suggests a close association between mediastinal lymph node metastasis and poor prognosis. In this study, the incidence of distant metastasis was also higher in patients with N2 lymph node metastasis (HR = 1.85, *p* = 0.003). The underlying cause of this survival difference is attributed to the pathophysiological mechanism of dual-pathway metastasis in lung cancer. Isolated pleural metastasis (M1a) initially primarily reflects direct tumor invasion and transcoelomic spread. If mediastinal lymph nodes remain uninvolved (N0/N1) during dissemination, this suggests that the tumor’s invasive potential has not yet compromised the complex lymphatic barrier network (15–17). For such patients, resection of the primary lesion and ipsilateral pleural metastasis achieves physical clearance of the tumor burden. Biologically, this procedure blocks the continuous release of cancer cells into the thoracic cavity, potentially yielding survival outcomes that surpass those of certain patients with locally advanced (stage III) disease (18, 19). Involvement of N2 lymph nodes indicates dissemination through the lymphatic system, suggesting that even if the local tumor is resected, circulating tumor cells are highly likely to be present in the bloodstream (20). Therefore, for patients with N2 lymph node metastasis, adjuvant therapy and follow-up monitoring should be strengthened postoperatively, and consolidation immunotherapy or targeted therapy may be considered if necessary (21). Furthermore, lymph node status should be accurately assessed preoperatively through imaging modalities and mediastinoscopy to optimize treatment decisions (22).

R0 resection is a critical determinant of long-term survival (23–25). Multivariate analysis identified R1 resection as an independent risk factor for OS (HR = 2.08, *p* = 0.024). The median OS for patients with R0 resection was 35.2 months, significantly longer than the 15.8 months for patients with R1 resection. In this study, the R0 resection rate was 86.22%, with approximately 13.77% of patients undergoing palliative surgery due to extensive tumor invasion.

During the follow-up period, disease recurrence occurred in 35 patients (20.95%). Of these cases, regional and local recurrences predominated (approximately 13.17%), while the incidence of distant organ metastases remained relatively low (7.78%). This distribution pattern underscores the “intra-thoracic localized” nature of isolated pleural metastasis in NSCLC and further supports the rationale for intensified surgical local control following effective conversion therapy (26).

Although this study suggests significant survival advantages of conversion surgery for stage M1a non-small cell lung cancer (NSCLC), the relatively small sample size (*n* = 167) limits the statistical power and generalizability of the findings. The limited sample size may mask clinically relevant effects or increase the risk of type II errors; therefore, the results of subgroup analyses should be interpreted with caution. Secondly, the retrospective design and lack of randomization introduce selection bias, including surgeon and patient selection effects. This approach may inadvertently skew the distribution of disease severity or adherence to treatment protocols, thereby limiting the representativeness of the study and potentially affecting outcome assessments. Finally, patients received heterogeneous systemic therapies, including first- to third-generation TKIs, various PD-1/PD-L1 inhibitors, and to varying degrees, antiangiogenic agents or platinum-based doublet chemotherapy (27–29). This diversity in therapeutic regimens means that while this study validates the effectiveness of conversion therapy broadly, it cannot definitively determine which specific molecular targeted drugs or immune-checkpoint inhibitor combinations can provide the best depth of downstaging and the longest overall survival (OS) benefit in the neoadjuvant setting for stage M1a disease (20, 30, 31). In summary, future multicenter, prospective, randomized controlled clinical trials (RCTs) are warranted to validate these findings. Quality of life studies should also be conducted to comprehensively assess the clinical benefits for patients.

## Conclusion

We retrospectively analyzed 167 patients with isolated pleural metastatic lung cancer who underwent surgical resection after effective conversion therapy; our analysis identified pathological type, molecular characteristics, and completeness of resection as key prognostic indicators. From a clinical perspective, patients who demonstrate a favorable response to conversion therapy, are candidates for R0 resection, and harbor driver gene mutations should be prioritized for surgical intervention.

## Data Availability

The original contributions presented in the study are included in the article/Supplementary material, further inquiries can be directed to the corresponding author.
